# ComPhy: prokaryotic composite distance phylogenies inferred from whole-genome gene sets

**DOI:** 10.1186/1471-2105-10-S1-S5

**Published:** 2009-01-30

**Authors:** Guan Ning Lin, Zhipeng Cai, Guohui Lin, Sounak Chakraborty, Dong Xu

**Affiliations:** 1 Digital Biology Laboratory, Informatics Institute, Computer Science Department and Christopher S. Bond Life Sciences Center, University of Missouri, Columbia, MO 65211, USA; 2 Department of Computing Science, University of Alberta, Edmonton, Alberta T6G 2E8, Canada; 3 Department of Statistics, University of Missouri, Columbia, MO 65211, USA

## Abstract

**Background:**

With the increasing availability of whole genome sequences, it is becoming more and more important to use complete genome sequences for inferring species phylogenies. We developed a new tool ComPhy, 'Composite Distance Phylogeny', based on a composite distance matrix calculated from the comparison of complete gene sets between genome pairs to produce a prokaryotic phylogeny.

**Results:**

The composite distance between two genomes is defined by three components: Gene Dispersion Distance (GDD), Genome Breakpoint Distance (GBD) and Gene Content Distance (GCD). GDD quantifies the dispersion of orthologous genes along the genomic coordinates from one genome to another; GBD measures the shared breakpoints between two genomes; GCD measures the level of shared orthologs between two genomes. The phylogenetic tree is constructed from the composite distance matrix using a neighbor joining method. We tested our method on 9 datasets from 398 completely sequenced prokaryotic genomes. We have achieved above 90% agreement in quartet topologies between the tree created by our method and the tree from the Bergey's taxonomy. In comparison to several other phylogenetic analysis methods, our method showed consistently better performance.

**Conclusion:**

ComPhy is a fast and robust tool for genome-wide inference of evolutionary relationship among genomes. It can be downloaded from .

## Background

The systematic classification of bacteria has been a long-standing problem because very limited morphological features are available. For a long time researchers could only group together similar bacteria for practical determinative needs [1]. Woese and collaborators initiated molecular phylogeny of prokaryotes by making use of the small subunit (SSU) ribosomal RNA (rRNA) sequences [2]. The ssu-rRNA trees [3] have been considered as the standard Tree of Life by many biologists.

Attempts to explicate the phylogeny of prokaryotes based on the ssu-rRNA have been by-and-large successful [3,4]. However, although such molecules have proved to be very useful phylogenetic markers, mutational saturation is a problem due to the restricted length and limited number of mutation sites [5]. Another well-known problem is that phylogenetic trees, constructed on single gene families may show conflicts [6] due to a variety of causes, such as LGT (Lateral Gene Transfer) [7], hybridization, lineage-sorting, paralogous genes [8], and pseudogenes [9]. With the increasing availability of whole genome sequences, methods using vast amounts of phylogenetic information contained in complete genome sequences are becoming more and more important for inferring species phylogenies. Because phylogenetic information extracted from whole genomes is based on the maximum genetic information, the resulting phylogenetic tree should be the best reflection of the evolutionary history of the species, assuming this history is tree-like [7,10]. Phylogenomics, i.e. using entire genomes to infer a species tree, represents the state of art for reconstructing phylogenies [11,12].

A relatively obvious approach to phylogenetic analysis of whole genomes uses multiple sequence alignments with certain evolutionary models [9,13]. However, the multiple sequence alignment strategy may not work for whole genomes and the evolutionary models may not always be applicable. Multiple sequence alignment could be misleading due to gene rearrangements, inversion, transposition and translocation at the genome level [7,8], unequal lengths of sequences, LGT, etc. On the other hand, reliable statistical evolution models are yet to be suggested for complete genomes.

To address these issues, Sankoff and Blanchette [18] defined an evolutionary edit distance as the number of inversions, transpositions and deletions/insertions required to change the gene order from one genome into another. Similar distance measures using rearrangement, recombination, breakpoint, comparative mapping and gene order have been extensively studied for whole genome phylogeny [14-21]. These approaches are computationally expensive, and in general do not produce correct results on events such as non-contiguous copies of a gene on the genome or non-decisive gene order.

Gene content was proposed as distance measure in whole genome phylogeny where "the similarity between two species is defined as the number of genes they have in common divided by their total number of genes" [22]. This idea was further extended to use lists of nucleotide segment pairs in comparison instead of lists of genes [23]. Such method fails when the gene contents of organisms are very similar, such as bacteria in closely related families, or chimerical genomes.

Overlapping gene information was also used to infer the genome phylogenies [24]. Overlapping genes are defined as pairs of adjacent genes of which the coding sequences overlap partly or entirely. Although overlapping genes have been shown to be a consistent and conserved feature across all microbial genomes sequenced to date [25], the limited amount of overlapping genes is usually not enough for evaluating a large number of genomes.

Some other methods infer phylogenies based on protein structural domain information [26-31], which considers LGT. However, they assume some proteome evolution models with lateral structural domain transfer events, which may not be accurate. Also, the method readjusts the protein structural domain graph each time when a LGT event is introduced, the complexity of model testing increases substantially when large number of lateral structural domain transfer events been assumed.

In this paper, we introduce a new tool 'ComPhy', which utilizes a robust and much less complex strategy, called 'Gene Composite Distance'. It combines different aspects of evolutionary relationships among genomes to produce a phylogenetic tree from a given set of whole genome sequences. We have applied this approach to 398 prokaryotic genomes, which were downloaded from NCBI [32]. More precisely, composite distance measure starts with an all-against-all pairwise genome comparison using BLASTP [33]. In the second step, a distance matrix is calculated from three components, i.e., GDD (Gene Dispersion distance), GBD (Genome Breakpoint distance) and GCD (Gene Content distance). This distance matrix is then fed to a distance-based algorithm, Neighbor-Joining (NJ) [34,35], using a third-party tool 'Phylip' [47] to produce a phylogenetic tree. In our current study, we do not consider LGT. Our goal is to have mathematically tractability and to develop a generalized phylogenetic distance model and a phylogenetic tree construction platform that can be easily applied to any species. Furthermore, using the completely sequenced genomes allows the construction of a phylogeny less sensitive to inconsistencies, such as LGT, unrecognized paralogy, and highly variable rates of evolution among different regions in a genome. The result phylogenetic trees are more representative of whole-genomes than those from single-gene trees.

## Methods

### Taxon selection

398 single-chromosome prokaryotic genome protein sequences were downloaded in the Fasta format from the NCBI [32] ftp server in September 2007. The physical gene location files of these genomes were also downloaded from NCBI in a tab-delimited format. We represent the species biological names as defined in Bergey's code [1]. For example, a lineage in the Bergey's Manual of Systematic Bacteriology or its online outline is abbreviated as B13.3.2.6.2 = Phylum BXIII (Firmicutes), Class III (Bacilli), Order II (Lactobacillales), Family VI (Streptococcaceae), Genus II (Lactococcus). Table 1 shows the taxon statistics of the 432 prokaryotic genomes, including 34 multi-chromosomal species that we do not consider in this paper.

**Table 1:** Taxon statistics of the 432 prokaryotic complete genomes

Phylum	C	O	F	G	S	str
A1	1	3	4	4	7	7
A2	8	9	12	18	23	23
A3	1	1	1	1	1	1

Subtotal 3	10	13	17	23	31	31

B1	1	1	1	1	1	1
B2	1	1	1	1	1	1
B4	1	2	2	2	3	4
B6	1	1	1	1	2	2
B10	1	3	3	8	15	19
B11	1	1	1	2	4	4
B12	5	33	53	99	157	208
B13	3	7	14	22	58	96
B14	3	9	15	16	31	35
B15	1	1	1	1	1	1
B16	1	1	2	3	7	11
B17	1	1	2	3	7	9
B19	1	1	1	2	2	2
B20	3	3	5	5	6	7
B21	1	1	1	1	1	1

Subtotal 15	25	66	103	167	296	401

Total 18	35	79	120	190	327	432

P = Phylum, C = Class, O = Order, F = Family, G = Genus, S = Species, str = Strain. A = Archaea and B = Bacteria.

In order to test the performance of our method, 9 datasets with different combinations of the whole 398 genomes are formed for different purposes. Dataset 1 is formed by 52 randomly selected species from the Bergey's taxonomy tree to test for robustness. We like to test the performance of our method on this broad range of species. Dataset 2 has 53 species, half of them are randomly picked from Archaea genomes (A in Bergey's code) and half are randomly picked from Baceteria genomes (B in Bergey's code). These 53 species from two major clades have a clear taxonomy structure with two clusters. Dataset 3 has 82 species, half of them are from Phylum B12 since half of 398 genomes are actually from B12 Phylum, and the other half is randomly selected from all the other types of genomes, i.e., half of them have a tight cluster and the other half are diverse. Dataset 4 includes all the 398 single chromosome genomes. Since many prokaryotes are from Phylum B12 and Phylum B13, therefore, we form datasets 5 and 6 from all the 181 Phylum B12 genomes and 96 Phylum B13 genomes, respectively, to test for the effects of the co-linearity of datasets on phylogeny construction. Dataset 7 is a union of datasets 5 and 6, again with two tight clusters of species. Dataset 8 has 165 prokaryotic species obtained from BPhyOG [24] in order to compare the performance between our method and the overlapping gene based phylogeny used by BPhyOG. Dataset 9, with 54 prokaryotic species was obtained from Deeds [31] for comparing performance between our method and the structural domain based phylogeny construction. Both dataset 8 or 9 contain a subset of the 398 complete prokaryotic data. We believe these 9 diverse datasets allow us to test the robustness of our method comprehensively.

### Identification of orthologs

The initial step in the phylogenetic analysis methods is to determine which genes are to be compared between species. Since the ultimate goal is determining the distance between every two genomes, intuitively we use pairwise orthology for every pair of genomes. So far there is no existing database containing the orthologous groups for all the genomes that we are studying.

Here, we define orthologs by performing an all-against-all BLAST between every pair of protein sequences for each pair of species. The reciprocal best BLAST hits are used to determine the list of orthologs between every pair of species. Additional filtering methods have also been applied to refine the list of orthologs between the pair of genomes, such as pairs of genes to be considered orthologs must satisfy BLAST hit with E-values below 10^-3 ^and sequence identity higher than 30%. The tests of variations of ortholog definition will be shown in the Result and Discussion section. In ComPhy, we also give the users the flexibility to apply their definitions of orthologs.

### Composite distance phylogeny

This strategy is to compute a distance between any two genomes *X *and *Y *based on the set of orthologs obtained in the previous step. This new systematic composite distance measurement takes into account both similarities and dissimilarities between every pair of genomes using their entire gene sets. ComPhy utilizes this composite distance formulation to determine the phylogeny for given genomes. The formulation will be discussed in three separated calculation steps, GDD, 'Gene Dispersion Distance', GBD, 'Genome Breakpoint Distance' and GCD, 'Gene Content Distance'.

### GDD (Gene dispersion distance)

The first component, GDD, is to quantify the extent of dispersion of orthologs from one genome to another. Our assumption is that the closer the two species in the evolutionary tree, the more similar the physical arrangements of corresponding orthologs are. The dispersion of orthologs from one genome to another can be seen as how far orthologs move away from their physical locations during evolution due to events such as rearrangement, recombination, insertion and deletion. The further the evolution distance of the two species, the more dispersed the orthologs between two species are. In other words, the distance separations of pairs of orthologs are more conserved between a pair of closely related genomes. To simplify the problem, we consider only pairs of orthologs that are right next to each other. The gene dispersion distance of an ortholog pair from genome A to genome B can be then formulated as

(1) $$d_{GDD}(A,B)=\frac{\sum l_{i,i+1}}{n^{2}}$$

where *l*_*i*, *i*+1 _is the distance separation between the *i*-th ortholog and the (*i+*1)-th ortholog of genome *B *in genome *A*. For example, if orthologs *b*_*i *_and *b*_*i*+1 _are next to each other on genome *B*, but their corresponding orthologs *a*_*x *_and *a*_*y *_in genome *A *are *l *orthologs apart (counting 1 ortholog separation as 1 distance unit), then the distance between this ortholog pair is *l*. *n *is number of orthologs between genomes *A *and *B*, which is the maximum dispersion distance. For normalization, one *n *is needed to normalize the size of a genome or the total number of orthologs (*n*). Another *n *is to normalize the dispersion distance against the maximum distance between two orthologs, which is *n *also. Thus, *n*^2 ^is needed for normalization. In fact, the normalization factors, such as *n*, *n*^2^, and *n*^3^, have been tested to see performances. Our study has shown that using *n*^2 ^as the normalization factor, in terms of range of dispersion distance and number of shared orthologs, has the optimal results.

Note, the gene dispersions, from *A *to *B *and from *B *to *A*, are not necessarily symmetric. We can define the dispersion distance measure in three different ways, namely, we can use the average *D*(*A*, *B*) = [*d*(*A*, *B*) + *d*(*B*, *A*)]/2 or use one of the two directions, either *D*(*A*, *B*) = *d*(*A*, *B*) or *D*(*A*, *B*) = *d*(*B*, *A*). Our study indicates that this directionality does have some impact on the overall performance and averaging over both directions produces better and more consistent results. Thus, the dispersion distance between genome A and B is defined as

(2) *D*(*A*, *B*) = [*d*(*A*, *B*) + *d*(*B*, *A*)]/2

**Figure 1:**
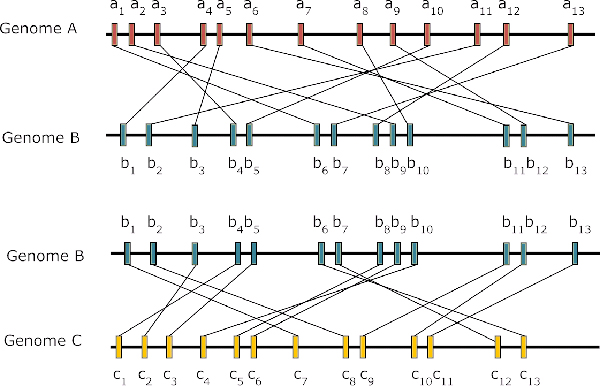
**Gene dispersions example between pairs of genomes**. The horizontal dark lines represent hypothetical genomes *A*, *B *and *C*. Each vertical box on the line is an ortholog and all orthologs are indexed according to their physical locations. The line connecting two boxes from one genome to another represents corresponding orthologs between two genomes.

Figure 1 shows two different dispersions of orthologs pairs between genome *B *and genome *A*, and between genome *B *and genome *C *as a hypothetical example. To calculate the distance between two genomes, we need to calculate each distance separation of pair of neighboring orthologs of one genome in another. In Figure 1, given there are 13 orthologs, the distance separation for orthologs pair of *b*_1_*b*_2 _is *a*_4_*a*_11_, so distance separation of *b*_1_*b*_2 _is $l_{b_{1}b_{2}}$ = *a*_11 _- *a*_4 _= 7, and distance separation of *b*_2_*b*_3 _is $l_{b_{2}b_{3}}$ = *a*_11 _- *a*_5 _= 6, etc. The total distance separations of all ortholog pairs *b*_*i*_*b*_*i*+1 _of genome *B *in *A *is $\sum l_{b_{i},b_{i+1}}$ = 66, and hence, *d*_*GDD*_(*B*, *A*) = 66/13^2 ^= 0.391 for GDD from genome *B *to *A*. Using same calculation, we can get *d*_*GDD*_(*A*, *B*) = 47/13^2 ^= 0.278, *d*_*GDD*_(*C*, *B*) = 38/13^2 ^= 0.225 and *d*_*GDD*_(*B*, *C*) = 37/13^2 ^= 0.219. We then get *D*(*A*, *B*) = 0.34 and *D*(*B*, *C*) = 0.22. As a result, genome *B *is closer related to genome *C *than to genome *A*. In other words, the ortholog pair distance separations are more conserved between *B *and *C *than between *A *and *B*.

**Figure 2:**
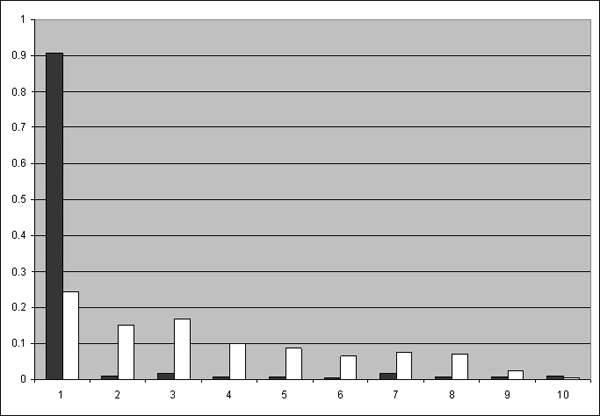
**Histogram of dispersion distance counts from *P. islandicum *to *P. aerophilum *and from *T. thermophilus *to *P. aerophilum***. We divide the range of dispersion distance between ortholog pairs into 10 bins on X-axis between *P. aerophilum *and *P. islandicum *and between *P. aerophilum *and *T. thermophilus*. The lower indexed bin contains shorter dispersion distance of ortholog pairs. The height of the bin represents the frequency of dispersion distance falling into the bin. Using *P. aerophilum *as the target genome, a black bar represents the frequency of the dispersion distance between genomes *P. aerophilum *and *P. islandicum*, and a white bar represents the frequency of dispersion distance between *P. aerophilum *and *T. thermophilus*.

We will also use a real example of three bacterial species, *Pyrobaculum aerophilum str. IM2 *(A1.1.1.1.3), *Pyrobaculum islandicum DSM 4185 *(A1.1.1.1.3), and *Thermus thermophilus HB27 *(B4.1.2.1.1) as an example to show the gene dispersion distance idea. By putting the dispersion distances of ortholog pairs, between *P. aerophilum *and *P*. *islandicum *and between *P. aerophilum *and *T. thermophilus*, in different distance bins, Figure 2 demonstrates the conservation of the dispersion distance of ortholog pairs between closely related species. *P. aerophilum *and *P*. *islandicum*, both belong to Thermoproteaceae family in Thermoprotei order, so in the figure the black bins show uneven distribution of frequencies and most dispersion distances are falling into the smallest distance bin. This result agrees with the experimental finding that these two species are highly similar in terms of contents of genes and overall genome organization [48]. In contrast, *T. thermophilus *is a member of Thermaceae family in Deinococci order, as a result the white bins show more evenly distributes of dispersion distances among distance bins.

### GBD (genome breakpoint distance)

**Figure 3:**
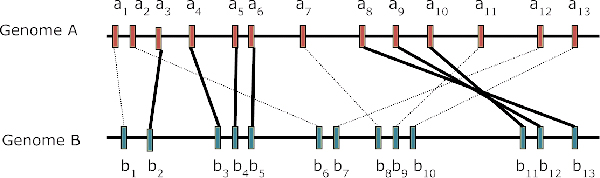
**Identification of Breakpoints**. The horizontal lines represent hypothetical genomes *A *and *B*. Each vertical box on the line is an ortholog and all orthologs are indexed according to their physical locations. The line connecting two boxes from one genome to another represents corresponding orthologs between two genomes. The solid lines connecting orthologs belong to some consecutive ortholog sets. The dotted lines connecting orthologs are orthologs pairs that are not in any consecutive ortholog set.

This distance transformation is based on the concept of breakpoints, where two sequence segments map consecutive intervals in one genome onto non-consecutive intervals in the other [18,40]. We simplified it by considering a breakpoint as where two ortholog sets map consecutively in one genome but not in the other. In other words, a breakpoint defined here is where the consecutive mapping of a set of orthologs between two genomes stops. Figure 3 gives a hypothetical example. There are two separated sets of consecutive orthologous genes and they are (*a*_3_*b*_2_, *a*_4_*b*_3_, *a*_5_*b*_4_, *a*_6_*b*_5_) and (*a*_8_*b*_13_, *a*_9_*b*_12_, *a*_10_*b*_11_), the consecutive mapping between genome *A *and *B *stops at positions *a*_2_*a*_3_, *a*_6_*a*_7 _and *a*_10_*a*_11 _in genome *B*. Therefore, there would be three breakpoints between genome *A *and genome *B*. Let *X*_*AB *_be the number of breakpoints between genome *A *and genome *B*, and *N*_*AB *_be the total number of orthologs between genome *A *and genome *B*. We then define a breakpoint similarity between *A *and *B *as

(4) $$D_{GBD}(A,B)=1-\frac{X_{AB}}{N_{AB}}$$

Using Figure 3 as an example, there are 3 breakpoints and there are 13 orthologs, and hence, *D*_*GBD*_(*A*, *B*) is 1–3/13 = 0.769.

### GCD (gene content distance)

The last component of composite distance is calculated using the idea of gene content [22] to show the similarity between two genomes. Here, we define the distance as

(3) $$D_{GCD}(X,Y)=1/(\frac{2*N_{c}}{N_{X}+N_{Y}})$$

where *N*_*c *_is the number of orthologs between *X *genome and *Y *genome, *N*_*X *_is the number of genes in genome *X*, and *N*_*Y *_is the number of genes in genome *Y*.

### Composite distance formulation

The composite distance used in this paper for genome distance calculation has three distance components described above, where they represent three different aspects of genomes. GDD describes conservation of relative physical separation distances of orthologs, where this conservation can be thought as evolution timestamps. GBD utilizes the ordering of genes between a pair of genomes. Although it has some correlation with the first component, GBD reflects more local synteny (such as micro-synteny) instead of large-scale genome rearrangement characterized by GDD. GCD shows the level of similarity shared from genome composition without considering gene locations. It acts as an adjustment for different sizes of genomes, which could be thought as a normalization factor. Preliminary experiments showed that they are all very informative (Table 2). Therefore, the composite distance is defined as following:

**Table 2:** Accuracy for different components combinations of our proposed distance method

Data sets	Number of species	GDD (%)	GCD (%)	GBD (%)	GCD*GDD (%)	GCD*GBD (%)	GDD*GBD (%)	GCD*GDD*GBD (%)
Dataset1	52	85.12	86.44	84.54	91.45	90.29	90.29	90.29
Dataset2	53	87.76	86.40	84.45	90.65	90.74	90.74	90.74
Dataset3	82	80.37	92.58	84.19	94.46	95.93	96.06	98.46
Dataset4	398	83.73	86.56	81.23	89.93	87.07	87.28	90.07
Dataset5	181	95.04	89.74	90.20	94.30	95.67	98.16	98.30
Dataset6	96	87.39	85.45	84.88	99.36	99.26	99.36	99.26
Dataset7	277	88.70	84.04	86.75	88.71	89.71	88.23	90.71
Dataset8	165	85.36	77.98	77.03	94.44	94.38	94.47	94.38
Dataset9	54	89.31	87.34	83.76	92.31	92.31	92.37	96.55

GDD = gene dispersion distance, GCD = gene content distance, GBD = gene breakpoint distance. GCD*GDD is the combination of two distance components and GCD*GDD*GBD is the combination of all three terms. All distances are logarithmically transformed.

(5) *D*(*X*, *Y*) = log*D*_*GDD *_+ log*D*_*GBD *_+ log*D*_*GCD*_

We apply logarithm to the formula for retaining precision of computing and correcting the saturation effects in sequence data [41]. By considering the three distances, we generalize the conservation of gene order into an accurate and robust measurement.

### Other genome distance measures

To compare with methods developed by other researchers, we also implemented several other distance measures for phylogenetic analysis.

#### (1) Overlapping gene phylogenetic distance

Overlapping genes (OG) [24] are defined as pairs of adjacent genes of which the coding sequences overlap partly or entirely. The distance between genomes *i *and *j *is defined as:

(6) $$D_{ij}=1-\frac{x_{ij}+x_{ji}}{2*\min(x_{i},x_{j})}$$

where *x*_*i *_is the number of OG pairs in genome *i *and *x*_*ij *_is the number of OG pairs in genome *i *that have their respective orthologs in genome *j*, and *vice versa *for other subscripts.

#### (2) Structural domain phylogenetic distance

The structural domain based distance method [31] uses the idea of Protein Domain Universe Graph (PDUG), a graph in which a nonredundant set of all known protein structural domains [42,43] are represented as nodes, and the structural similarity between domains is used to define edges between them. The distribution of edges per node in the graph was shown markedly different from random graph [44]. By combining with information of probability of LGT events, a similarity distance between species could be constructed. For example, degree distribution is calculated by comparing domain graph against known PDUG as follows:

(7) $$p_{N}(k)=\sum_{s=k}^{MaxkN_{0}}\left(\begin{array}{c} s\\ k \end{array}\right)\left(\frac{N}{N_{0}}\right){\left(1-\frac{N}{N_{0}}\right)}^{s-k}$$

where $Maxk_{N_{0}}$ is the degree of the maximally connected node in the underlying graph (such as PDUG) with *N*_0 _nodes and a species graph with *N *nodes.

LGT is modeled as the movement of a node from a proteome in which that node exists into a proteome in which it does not. A transfer does not remove the node from the “donor” organism, but it may replace (thus, “erase”) one of the nodes in the acceptor organism to preserve the proteome size. The donor and acceptor organisms are chosen randomly, and the transferred node is chosen randomly from the set of nodes in the donor proteome that do not exist in the acceptor proteome. The acceptor node that is replaced is also chosen at random.

#### (3) CCV/CV-based phylogenetic distance

Gao et al. [45] used all the string appearance frequencies (strings of length k) to represent each genome, that is, each whole genome can be regarded as a high-dimension vector, where each vector component is the frequency of a particular combination of nucleotides (A, T, C, G). Then the pairwise distance between two genomes can be calculated as the Euclidean distance between the corresponding two vectors. Wu et al. [46] extended this idea to use all the string appearance frequencies to define a CCV-based phylogenetic distance. In this distance, strings of length from 1 to k are all employed.

### Phylogenetic inference

All of methods mentioned above, including composite distance method, were used to generate a distance matrix between all pairs of genomes. Phylogenetic trees were then generated using the Neighbor-Joining [34,35] algorithm in Phylip (version 3.67) [47] (likelihood based approaches will be applied in future studies).

### Performance measurement

**Figure 4:**
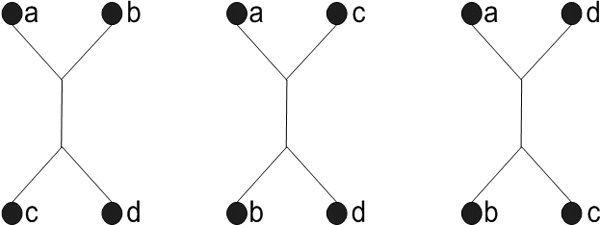
**Three possible quarter topologies**. Given four taxa (a, b, c, and d), there are only three unique ways to connect the taxa as terminals.

There is no official standard for prokaryotic taxonomy. However, it is widely believed by microbiologists that the classification scheme in Bergey's Manual of Systematic Bacteriology [1] is the best approximation available. To measure the performances of a distance method, we calculated the percentage of agreed quartet topologies between the tree created by the method and the tree from the Bergey's taxonomy. A quartet topology is a subtree structure of the subset of 4 taxa (called a quartet). Given a quartet of taxa, a, b, c, and d, there are 3 possible ways to connect the taxa as terminals (Figure 4). Note that, the tree from Bergey's taxonomy is not a binary tree; therefore, one node may have more than two children. As a result, some of the quartets do not have any of the three quartet topologies in Figure 4. We call a quartet is resolved if no more than three of taxa share the same parents. To measure the performance of our method, we will collect agreed quartets in which the quartets have the same topology between the tree from Bergey's taxonomy and a binary phylogenetic tree. The accuracy is percentage of the agreed quartets.

## Results and discussion

### Variance of orthlog definition

To test the robustness of our ortholog definition, different variations of E-value cut-offs and sequence identities have been selected for performance evaluation. Our results showed that E-value cut-offs lower than 10^-3 ^do not have significant effects on the results. This is probably because the reciprocal Blast hit process would ensure the majority of the homolog selections as long as E-values are small enough. However, different selections of percentage identities have some impacts on the performance. A very high percentage identity would be too stringent to obtain enough number of orthologs, while a very low percentage identity would have too many false positive ortholog pairs. We used dataset 9 as representative dataset, described in the Method section, for testing orthologs selection with E-value below 10–3 and percentage identities of 10%, 20%, 30% and 40%. Accuracies of 76%, 82%, 89% and 85% were obtained using GDD method, respectively. Therefore, we selected the E-value cut-off at 10–3 and percentage identity of 30% for the optimal ortholog definition.

### Comparison to single gene trees

**Figure 5:**
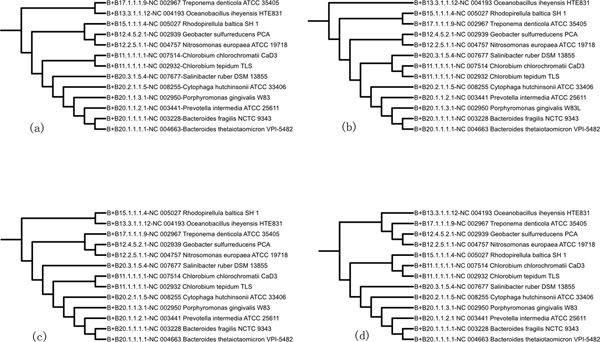
**Phylogenetic trees based on different gene selections**. (a) Phylogenetic tree of 13 bacterial species based on CTP synthase (*pyrG*) affiliates with *Bacteroidates*; (b) phylogenetic tree of 13 bacterial species based on *glyA *affiliates with *Chlorobi*; (c) phylogenetic tree of 13 bacterial species based on Chaperonim Hsp 60 (*groEL*) affiliates with the superphylum *Bacteroidates-Chlorobi*. (d) phylogenetic tree of 13 bacterial species based on the whole-genome gene sets.

Although attempts to explain the phylogeny of prokaryotes based on the ssu-rRNA have been quite successful [3,4], a well-known problem associated with this type of single-gene approach is that the evolutionary history of any single gene may differ from the phylogenetic history of the whole organism from which the corresponding molecule was isolated. We were able to obtain single-gene phylogenetic trees of 13 bacterial species [51] for comparing with our method. Figure 5 shows three different trees based on single-gene selection and one tree based on the whole-genome gene sets using our method. Using the accuracy measurement described in the Method section, accuracies of 88%, 81% and 83% are obtained for tree (a), (b) and (c), respectively, while tree (d) based on the whole-genome gene sets has a significantly higher accuracy of 91%. As an example, in tree (b) and (c), *Salinnibacter rubber*, which is a member of Bacteroidetes (B20), is placed outside its own phylum towards the protebacteria phylum (B12). In tree (d), *Rhodopirellula baltica *is placed closer to phyla Bacteroidetes (B20) and Chlorobi (B11), which is correct [52]. Huerta-Cepas *et al*. [53] found degrees of topological variations among single-gene phylogenies were much greater than previously thought. Their conclusions, although based on eukaryotes, may be applicable to the whole tree of life, and are probably even more important to the prokaryote phylogeny given more LGTs in prokaryotes.

### Composite distance as optimal distance measurement

We used 9 datasets, described in the Method section, for performance evaluations. In comparison to several other phylogenetic analysis methods, our composite distance method showed consistently better performance (see Tables 2 and 3). We have achieved above 90% accuracy comparing to Bergey's taxonomy system for all the selected datasets.

**Table 3:** Accuracy comparison between our composite distance method and other methods

Data sets	Number of species	GCD (%)	OG (%)	CCV (k <= 5) (%)	CCV(k = 5) (%)	SDD (%)	Composite Distance (%)
Dataset1	52	86.44	83.93	87.82	88.29	NA	90.29
Dataset2	53	86.40	85.49	86.27	87.92	NA	90.74
Dataset3	82	92.58	84.35	95.97	91.54	NA	98.46
Dataset4	398	86.56	85.52	79.03	78.86	NA	90.07
Dataset5	181	89.74	80.34	87.19	87.19	NA	98.30
Dataset6	96	85.45	87.22	99.00	99.07	NA	99.26
Dataset7	277	84.04	81.89	83.28	83.19	NA	90.71
Dataset8	165	77.98	87.87	85.20	82.78	NA	94.38
Dataset9	54	87.34	88.27	91.39	91.47	81.57	96.55

GCD = gene content distance, OG = overlapping gene distance, SDD = structural domain distance.

Table 2 shows the performance results of 7 different combinations of three composite distance components using the 9 datasets based on performance evaluation method described in the Method section. Table 3 compares the performances of 5 different methods including our proposed method. The accuracy is defined as the percentage of agreed quartets between the tree created by a distance method and the tree from the Bergey's taxonomy system. Due to the complexity of obtaining accurate LGT events and protein structural models for all the species to apply the Structural Domain method on the genome distance calculation, we could not obtain the genome phylogeny trees for all the 9 datasets except dataset 9, whose result can be directly downloaded from [31].

Results from Tables 2 and 3 suggest the optimal distance calculation is the composite distance, which combines the advantages of GDD, GCD and GBD. We use dataset 9 by Deeds [31] for detailed discussion. Dataset 9 consists of 8 Archaea (A2) species, 1 Bacteria Aquificae (B1) species, 1 Bacteria Thermotogae (B2) species, 2 Bacteria Cyanobacteria (B10) species, 21 Bacteria Proteobacteria (B12) species, 15 Bacteria Firmicutes (B13) species, 5 Bacteria Actinobacteria (B14) species, and 1 Bacteria Fusobacteria (B21) species. In this dataset, *Thermotoga maritima *(B2.1.1.1.1), a rod-shaped bacterium belongs to the order Thermotogales, contains 1,877 predicted coding regions, but it has only about 110 genes that have orthologs in the genomes of other thermophilic Eubacteria and Archaea. Completion of its genome has revealed a high degree of similarity with Archaea in terms of contents of genes and overall genome organization, where almost one quarter of the genome is Archaea in nature, instead of other bacterial phylum. Conservation of gene order between *T. maritima *and the Archaea species in many of the clustered regions suggests that LGT may have occurred between thermophilic Eubacteria and Archaea [36]. When classifying this species, our composite distance method moves *T. maritima *closer to the Archaea clade on the tree, which reflects this biological property. In contrast, other methods, such as CCV method and overlapping gene method, put *T. maritima *closer to either the Proteobacteria (B12) clade or the Firmicutes (B13) clade on the phylogenetic tree. Out of these three methods that do not model LGT events specifically, the composite distance method still demonstrates good sensitivity of the proposed composite distance method.

By comparing different trees generated from different distance measures, we found that GDD and GBD are more sensitive on deeper-lever of the trees. However, GCD is more accurate on higher levels, such as clades of the tree. For example, the gene content method puts five species of Diplococci (class) of Fiemicutes (B13) together with its neighboring class Bacilli of Fiemicutes, while GDD mis-classifies those five species as members of the Proteobactria (B12) clade. As we can see from Table 2, by combining all three distance components together as the composite distance measure, the advantages of individual components are also combined together to achieve the optimal results. Other methods, such as structural domain method, position Archaea at a higher level phylum than Bacteria phylum on the phylogeny tree, which contradicts the taxonomy where they are at the parallel levels on the tree. In contrast, our composite distance method is consistent with the taxonomy. A possible reason could be the consideration of LGT events, as the structural domain method overly emphasizes the role of LGT genes from Archaea to Bacteria. The overlapping gene method however, has problem of using limited information to estimate the distance. Overlapping genes are conserved, but they represent a small number of genes in the genome and hence, the statistical errors may be large. This is shown by mis-classifying *Pyrococcus furiosus *(A2.6.1.1.3) into clade of Bacilli (B13), instead of Archaea (A2) using the overlapping gene method.

It is interesting to note that GBD method by itself performed poorly for large-scale comparison of prokaryote genomes, which is in accordance with the commonly held view that breakpoint methods lead to reliable results only if the genomes are sufficiently co-linear, such as in datasets 5 and 6.

### Efficiency comparison

Besides comparing the accuracies of different distance measures, we also consider computational efficiency. Most whole-genome phylogeny construction methods, including ours, require a process of defining orthologs through time-consuming BLAST. Assuming there are *m *genomes and each genome has roughly ~*n *orthologs, then the complexity for reciprocal BLAST hits in ortholog identifications would be *O*(*m*^2^*n*^2^). Excluding this process, our method, which computes in linear time, takes much less computing time than other methods, especially the breakpoint distance measure [18]. Structural domain method, in another way, considers LGT events. It approximates the genome distance by continuously readjusting the protein structural domain graph when applying each LGT event (see the Method section for more details), and the running time could easily take up to hours if not days for a large genome data set. The CCV method, although not requiring a process of defining orthologs, considers every possible string of length up to k for whole genome sequences. This method requires even higher computational resources in terms of memory and CPU cycles. Overall, the composite distance measure shows not only the higher accuracy but also fast speed.

### Further discussion

Given pairs of prokaryotic species, there could be situations where one genome is essentially a subset of another much larger genome. For example, in our datasets this is true of *Buchnera aphidicola *genome, which is essentially a subset of the *Escherichia coli *genome, with approximately 14% the size of it [49]. Shared genome sequences could make two different genomes seem to be more closely related than they actually are. We have tried to model this case by modifying the GCD formula since this method uses all the genes in genome pair as normalization factor, not just the orthologs. We used the smaller genome gene set size instead of summing two genome gene set sizes for normalization. This modified formula would consider the similar segments of genome at most once. However, the performance of this modified GCD formula decreased significantly from around 85% to 60% for most of the datasets. Given that the situation where one genome is part of another genome is rare, it appears that considering this in our distance calculation lost the generality of the method.

Although all our trees would be generated as binary trees, or phylograms, with two leaf species for each node and they are hard to compare to the taxonomy, which is usually not binary, we find our results are consistent with most of the taxonomy in Bergey's system based on the percentage of agreed quartet topologies. Nevertheless, we still mis-classify some species on the tree. For example, *Treponema pallidum *in class Spirochaetes (B17.1) is placed as a sib of the class phylum Diplococci (B14.1), which does not agree with current classifications. This may be because the genome *T. pallidum *has high number of LGTs, which is as high as 32.6% [37]. It would be hard for the current method to deal with such an extreme. Other cases, such as chimerical genomes [37] or paralogous genes, are not modeled in this study, but could have misleading effects on our classifications. Chimeric genomes, which could have happened due to LGT, would produce false lineage for the interested genomes. Paralogous genes would artificially shorten the distance between two genomes if there are many paralogs. Future developments of the tool would include events of LGT, gene copy number, and conservation of overlapping genes, as well as exclude genes with abnormal evolution rates. We can see that ComPhy provides a framework for incorporating more relevant biological aspects for distance measurement.

## Conclusion

ComPhy, a stand-alone phylogeny construction tool, provides a robust and easy-to-use tool for biologists. It does not require multiple sequence alignment and is fully automated. ComPhy implements a composite distance method, which does not depend on any type of evolution models in calculating the distance between two genomes besides the protein sequences and gene physical locations. It allows users to infer phylogenies for any set of genomes of interest to study their evolutionary relationships by either generating a phylogram tree or a Newick format tree file for further study. Although the tool is built for complete-genome gene sets phylogeny, users can provide pre-defined ortholog sets to build the phylogeny according their criteria. The process takes less than a minute from given protein sequence files and protein location files to the outputs of trees for hundreds of species if excluding the BLASTP for generating orthologs. Although in the current stage of the application, our method works only for species with single chromosome, we will extend ComPhy to study eukaryotic genomes with improved methods working on multi-chromosomes. We believe this is a timely development as the whole-genome phylogeny becomes dominant with the arrival of more complete genome sequences, especially from the meta-genomic analyses of microbial communities [38,39].

## Availability

ComPhy, all the datasets (including genome sequences, gene location files and Bergey's code), and the phylogenetic trees generated in this study are available at .

## Competing interests

The authors declare that they have no competing interests.

## Authors' contributions

GNL carried out the phylogeny constructions and drafted the manuscript. ZC and GL designed the datasets and provided the performance evaluation codes. SC provided some ideas and formulations. DX conceived and coordinated the study. All authors read and approved the final manuscript.
